# Supplementary material for: A Critical View of the Application of the APEX Software (Aqueous Photochemistry of Environmentally-Occurring Xenobiotics) to Predict Photoreaction Kinetics in Surface Freshwaters
Source: Molecules. 2019 Dec 18;25(1):9. doi: 10.3390/molecules25010009 (PMC7017383; doi:10.3390/molecules25010009)
Supplement: Supplementary file 1 [file molecules-25-00009-s001.zip › 3.2.4_gcc-4.4.0.zip!/3.2.4_gcc-4.4.0/3.2.4_gcc-4.4.0/doc/octave/html/interpreter/Graphics-backends.html]

Graphics backends - Untitled


Previous: Object Groups,
Up: Advanced Plotting


---

#### 15.2.9 Graphics backends

— Function File: **backend** (name)  
— Function File: **backend** (hlist, name)  
> Change the default graphics backend to name. If the backend is
> not already loaded, it is first initialized (initialization is done
> through the execution of `__init_`name`__`).
>
> When called with a list of figure handles, hlist, the backend is
> changed only for the listed figures.
>
>
>
> **See also:** available\_backends.

— Built-in Function: **available\_backends** ()  
> Return a cell array of registered graphics backends.

- Interaction with gnuplot
